# Exploring the Role of HSD17B2 in Colorectal Cancer Through Bioinformatic Analysis: Preliminary Insights for Prognostic Evaluation

**DOI:** 10.3390/ijms27177614

**Published:** 2026-08-25

**Authors:** Ana Belen Diaz-Ruano, Eliana Gomez-Jimenez, Alba Hidalgo-Ramirez, Giselle Xavier-Reis, Nieves Casillas-Ruiz, Noelia Garcia-Mascaraque, Alfonso Rubio-Navarro, Maria Paz Zafra, Juan Antonio Marchal, Manuel Picon-Ruiz

**Affiliations:** 1 Instituto de Investigación Biosanitaria ibs.GRANADA, University Hospitals of Granada, University of Granada, 18100 Granada, Spain; 2 Biopathology and Regenerative Medicine Institute (IBIMER), Centre for Biomedical Research (CIBM), University of Granada, 18100 Granada, Spain; 3 Department of Human Anatomy and Embryology, Faculty of Medicine, University of Granada, 18016 Granada, Spain; 4 Excellence Research Unit “Modeling Nature” (MNat), University of Granada, 18100 Granada, Spain; 5 Department of Biochemistry and Molecular Biology III and Immunology, Faculty of Medicine, University of Granada, 18016 Granada, Spain

**Keywords:** colorectal cancer, HSD17B, estradiol, estrone, detection

## Abstract

Colorectal cancer (CRC) is the third most commonly diagnosed cancer and the second leading cause of cancer-related mortality worldwide. Although screening has reduced CRC in older adults, cases in younger individuals are rising, highlighting the need for early biomarkers. Emerging research highlights the role of estrogen metabolism in CRC progression, with enzymes such as hydroxysteroid (17-beta) dehydrogenase (HSD17B) being increasingly implicated. In this study, we performed a bioinformatics analysis using publicly available datasets, including The Cancer Genome Atlas Colon Adenocarcinoma (TCGA-COAD) cohort and two independent Gene Expression Omnibus (GEO) cohorts (GSE40967 and GSE41258), to investigate the role of HSD17B enzymes in CRC. Our results suggest that HSD17B2 is frequently downregulated in precancerous lesions and early-stage CRC, which may contribute to elevated estradiol levels and a tumor-promoting microenvironment. In advanced stages, higher HSD17B2 expression levels are associated with poorer survival outcomes in retrospective cohorts. Other HSD17B enzymes also exhibit significant expression changes, further complicating the hormonal landscape of CRC. In addition, estrone, traditionally considered a weaker estrogen, emerges as a potential driver of CRC progression. Our in-silico analyses indicate that HSD17B2 and HSD17B11 warrant further investigation as candidate biomarkers for distinguishing CRC from benign and precancerous conditions, with the combination showing strong discriminatory power in Receiver Operating Characteristic (ROC) analyses. Overall, these findings highlight the potential role of estrogen metabolism in CRC and suggest that HSD17B enzymes may hold value as candidate prognostic and diagnostic indicators, though their clinical utility remains hypothetical at this stage. Experimental and clinical validation is strictly required to confirm these in silico observations and to clarify their mechanisms in CRC.

## 1. Introduction

Colorectal cancer (CRC) is the third most common cancer diagnosed and the second leading cause of mortality worldwide, with an estimated 1.93 million new cases and almost 1.0 million deaths in 2022 [1]. CRC affects both men and women, and in recent years, there has been a troubling increase in its incidence among younger populations [2,3]. Although CRC incidence and mortality rates have been decreasing in older adults due to improved screening programs [4,5], the rising prevalence among younger individuals highlights the urgent need for the development of early diagnostic biomarkers that can detect the disease at a more treatable stage.

One of the emerging areas of research in CRC focuses on the role of sex hormones, particularly estrogens, in the development and progression of the disease. The differential incidence of CRC in men and women has often suggested the involvement of sex hormones as a contributing factor [6,7,8]. While estrogens have been extensively studied in cancers including breast, ovarian, and prostate cancer [8,9,10], their specific role in CRC has only recently begun to garner more attention. Growing evidence suggests that estrogens may influence CRC progression through their interactions with cellular receptors involving various signaling pathways [11,12,13], opening new possibilities for understanding the biological mechanisms underlying CRC and identifying potential biomarkers for early detection [6,10].

Estrogens are known to regulate critical cellular processes such as cell proliferation, apoptosis, and DNA repair mechanisms [14,15]. Those functions are central to cancer development and progression. Studies have indicated that postmenopausal women, who experience a natural decline in estrogen levels, have an increased risk of CRC compared to premenopausal women, suggesting a potential protective role of estrogens against CRC [6,8,9,10,16].

One key enzyme, aromatase (CYP19A1), which converts androgens into estrogens, plays a central role in regulating local estrogen levels in tissues, including the colon [17,18]. CYP19A1 activity has been recently linked to/studied in CRC development and progression, particularly in relation to sex differences, offering preliminary insights into how estrogen imbalances contribute to cancer risk [17,18].

Another group of enzymes crucial for regulating estrogen and androgen activity is the hydroxysteroid (17-beta) dehydrogenase (HSD17B) family. This family consists of 14 isoforms, each with distinct tissue expression patterns and functions. These enzymes facilitate the interconversion between active and inactive forms of steroid hormones, such as estradiol and estrone or testosterone and androstenedione, playing a critical role in maintaining hormonal balance [19,20,21].

While the HSD17B family has been extensively studied in hormone-dependent cancers like breast and prostate cancer [21,22], its role in CRC remains not well understood. Emerging evidence suggests that some members of this family may influence colorectal carcinogenesis by regulating local estrogen and androgen levels in the colon [13,23,24]. The dysregulation of these enzymes could lead to hormonal imbalances that promote tumor growth and progression. Understanding how the HSD17B enzymes function in CRC could uncover the hormonal mechanisms driving the disease, potentially identifying new biomarkers for early diagnosis and prognosis [9,10,17].

In this study, we aim to investigate the expression patterns of the HSD17B family and their association with clinical parameters. By focusing on the role of estrogens, estrogen-related pathways and related enzymes in colorectal carcinogenesis, we hope to contribute to the discovery of early diagnostic biomarkers that could ultimately enhance prevention, detection, and treatment strategies in both men and women.

## 2. Results

### 2.1. Expression and Clinical Relevance of CYP19A1 in CRC

A pivotal enzyme in estrogen metabolism, extensively studied in various estrogen-related cancers, is CYP19A1. Therefore, we first evaluated alterations in transcriptional expression levels of this ubiquitous enzyme through an in silico analysis using The University of Alabama at Birmingham Cancer data analysis Portal (UALCAN) database. This analysis included a sample size of 114 controls and 1097 tumor samples. We compared colon adenocarcinoma with their respective healthy controls across multiple parameters.

**Figure 1:**
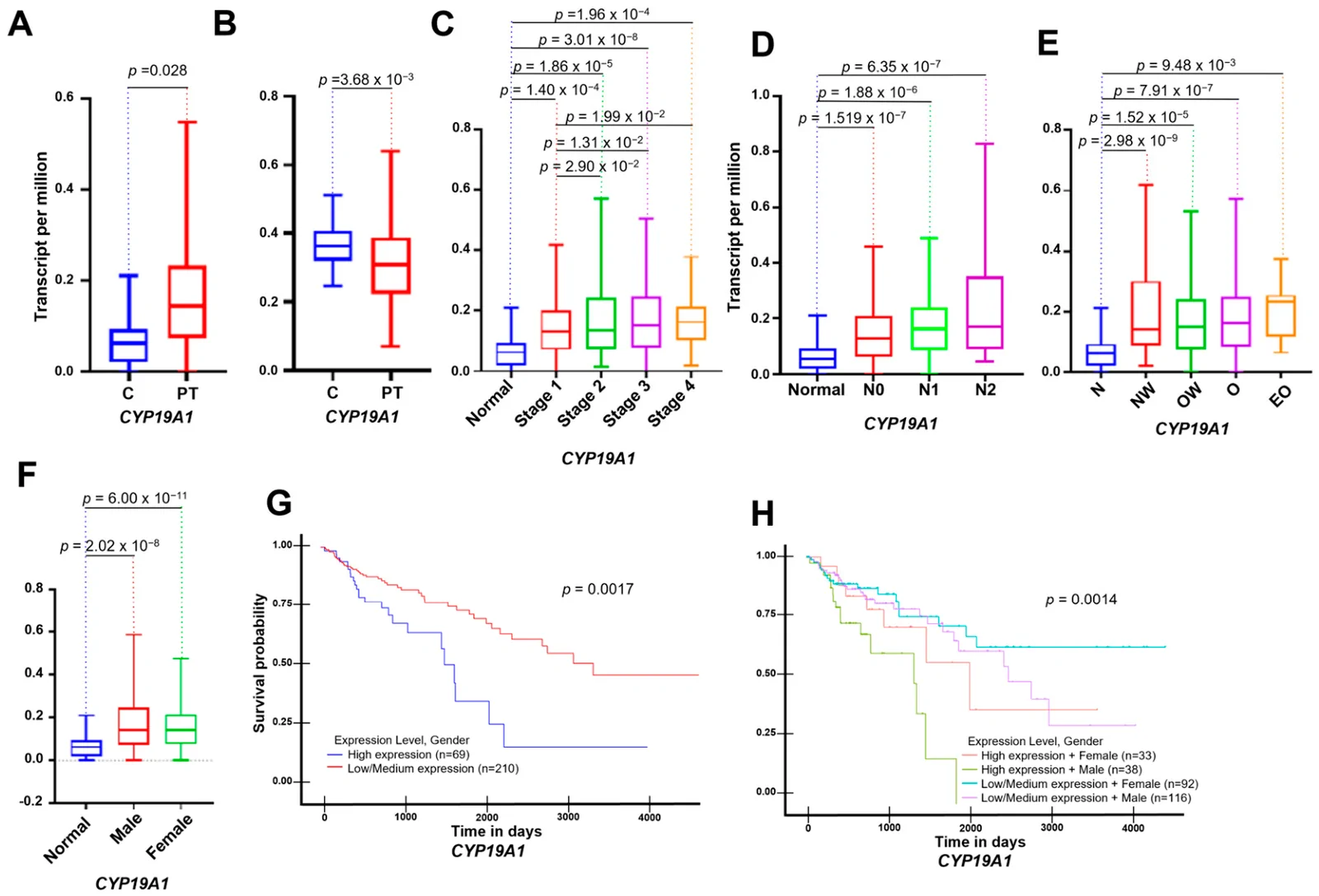
Expression profile, Promoter Methylation and Survival Analysis of CYP19A1 in Colon Adenocarcinoma. (**A**) Comparison of *CYP19A1* mRNA expression levels (expressed as transcript per million (TPM)) in primary tumor (PT, *n* = 1097) vs. control (C, *n* = 114). (**B**) *CYP19A1* promoter methylation levels in primary tumor vs. control. (**C**–**F**) Comparison of mRNA *CYP19A1* expression levels depending on (**C**) the stage of CRC, (**D**) nodal metastasis status, (**E**) body max index BMI (Normal Weight (NW), Overweight (OW), Obese (O) and Extreme Obese (EO)) and (**F**) sex. (**G**,**H**) Kaplan–Meier overall survival curve depending on *CYP19A1* expression levels for (**G**) all CRC patients or (**H**) segregated by sex. Statistical significance for expression and methylation analyses was calculated using UALCAN’s built-in Student’s *t*-test, with exact *p*-values provided for each comparison. Survival analyses were performed using the Kaplan–Meier method with log-rank test.

As shown in Figure 1A,B, transcript per million (TPM) levels of CYP19A1 were significantly elevated in tumor samples compared to healthy controls. Consistent with these expression trends, promoter methylation levels showed a significant decrease in tumor tissues relative to controls (Figure 1B). These correlative findings suggest a potential epigenetic mechanism underlying the upregulation of CYP19A1 in colon adenocarcinoma, though functional validation remains necessary.

Further analysis of patient data based on tumor stage, nodal metastasis and overweight status (Figure 1C–E) demonstrated a consistent upward trend in *CYP19A1* expression levels. Notably, increased aromatase expression correlated with higher stages of nodal metastasis, advanced tumor grading, and obesity status, reaching statistical significance. These in silico observations point to a potential link between *CYP19A1* levels, tumor progression and host factors such as obesity. In line with these findings, the survival analysis revealed that high levels of *CYP19A1* expression were significantly associated with poorer survival outcomes (Figure 1G). While high *CYP19A1* levels were observed across both male and female cohorts (Figure 1F), the inverse association with survival appeared more pronounced in men (Figure 1G,H), suggesting possible sex-related differences in outcome. However, this correlative finding should be interpreted with caution given the underlying biological differences between men and women. Overall, these results warrant further experimental research into the mechanisms that may contribute to this disparity and their potential implications for personalized treatment strategies.

**Figure 2:**
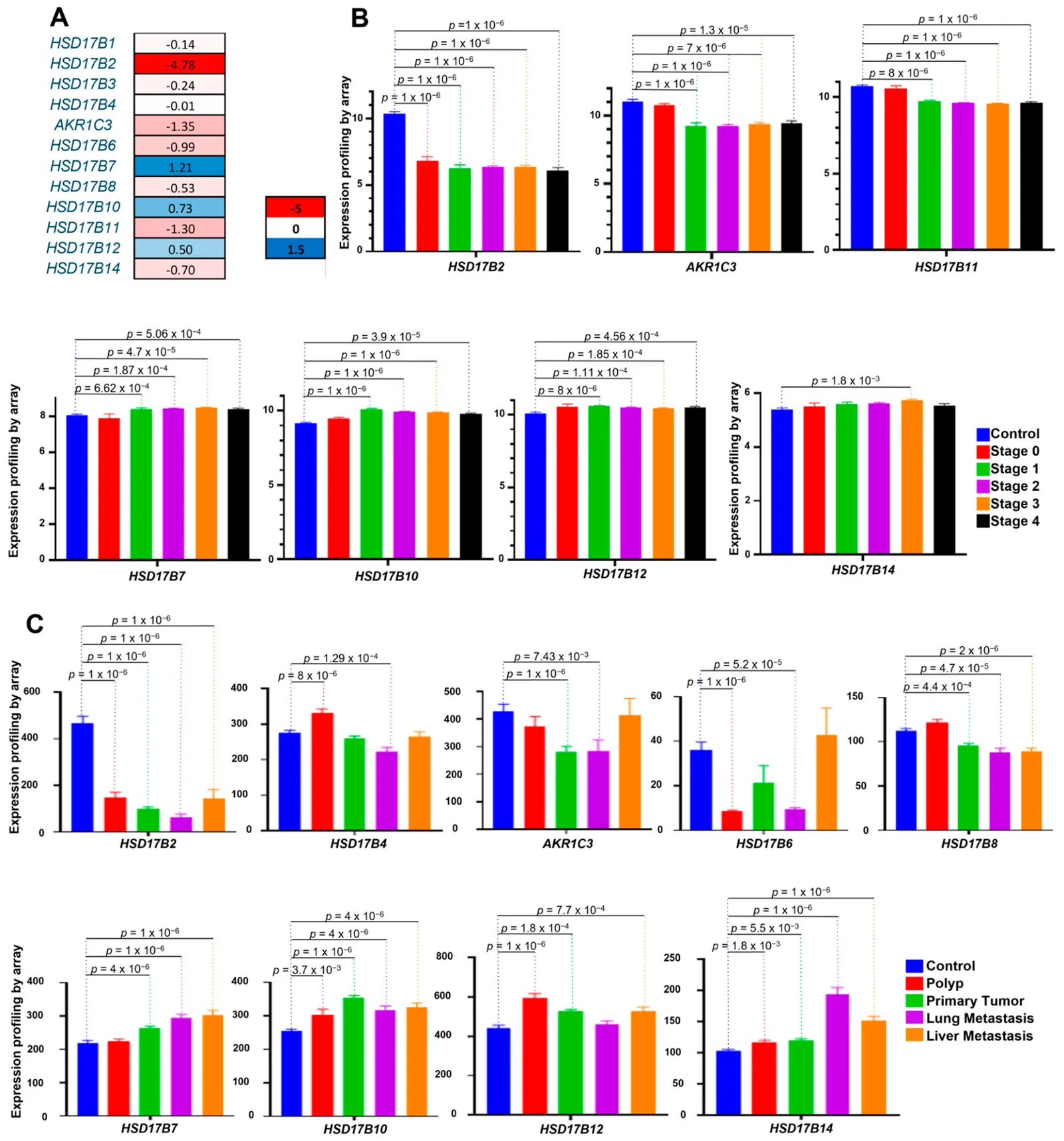
Transcriptional expression landscape of the HSD17B family across CRC progression datasets. (**A**) Heatmap representing the log2 fold change in TPM values for tumor samples (*n* = 1097) compared to controls (*n* = 114), derived from the UALCAN database. (**B**) Expression levels of HSD17B family members across control samples (*n* = 19) and CRC stage progression (Stage 0 to Stage IV, *n* = 566) from GEO dataset GSE40967. (**C**) Expression profiles of HSD17B family enzymes across healthy controls (*n* = 54), adenomatous polyps (*n* = 49), primary CRC tumors (*n* = 186) and distant metastatic sites (lung, *n* = 20, liver, *n* = 47) from GEO dataset GSE41258. Statistical significance between groups was evaluated using Student’s *t*-test with exact *p*-values provided for each comparison.

While CYP19A1 controls initial estrogen biosynthesis, local active hormone availability is ultimately fine-turned downstream by the HSD17B enzyme family, which directly regulates the interconversion between estrone and estradiol. Given its key physiological role, HSD17B2 emerges as a critical node in local steroid metabolism. Therefore, following our initial CYP19A1 findings, we extend our in silico analysis to systematically evaluate the HSD17B family, with a tailored focus on HSD17B2, across transcriptional, methylation and prognostic profiles (Figure 2).

### 2.2. Differential Expression of HSD17B Enzymes in CRC

The analysis of the HSD17B enzyme family in CRC reveals significant expression alterations across different stages of disease progression. Among these enzymes, HSD17B2 stands out due to its marked dysregulation.

Figure 2A shows a heatmap analysis representing the Log2(Fold Change) of tumor samples compared to controls of each HSD17B family member, using the TCGA-COAD cohort available through UALCAN (114 controls and 1097 tumor samples). To assess the reproducibility of these findings, transcriptional expression patterns were further evaluated in two independent Gene Expression Omnibus (GEO) cohorts. Figure 2B shows the analysis of GSE40967 (19 controls and 566 tumor samples), while Figure 2C corresponds to GSE41258 (54 control samples, 49 polyps, 186 primary tumors, 20 lung metastases and 47 liver metastases).

As indicated in the Supplementary Materials, the analyzed enzymes share a common function in mediating the interconversion between estradiol and estrone. Enzymes such as HSD17B1, AKR1C3, HSD17B7, and HSD17B12 are involved in the conversion of estrone to estradiol, potentially increasing local estradiol levels in the tumor. In contrast, enzymes such as HSD17B2, HSD17B4, HSD17B6, HSD17B8, HSD17B10, HSD17B11, and HSD17B14 convert estradiol to estrone, increasing local estrone levels. The differential regulation of the set of enzymes that synthesize estradiol, as well as those that synthesize estrone, highlights the complexity of hormonal regulation in CRC. These initial in silico findings suggest that changes in estrogen metabolism in CRC could warrant further investigation regarding their potential role in disease progression.

### 2.3. HSD17B2 Downregulation in Early Stages Suggest Potential Diagnostic Relevance

In Figure 2B, the negative and positive correlations between the different enzymes and the stages of the disease are shown. We can see how the enzymes HSD17B2 and AKR1C3 undergo the most significant expression changes, although, interestingly, both enzymes perform completely opposite functions. Furthermore, the expression changes are practically similar between the enzymes shown. It is worth noting that, once again, we find enzymes that have the same positive correlation with the tumor stage, although they have different functionalities. Remarkably, among all, the enzyme HSD17B14 shows an upward positive expression when comparing CRC cases with controls (Figure 2C).

When we compare enzyme expression across disease progression, from polyps to metastatic sites (Figure 2C), the data highlight that the noteworthy downregulation of HSD17B2 in early-stage CRC. HSD17B2 expression is significantly reduced in both polyp and primary tumor samples, with no recovery in metastatic samples. This early and sustained reduction in expression suggests its potential utility as a candidate marker for further study in early stages.

The constant decrease observed in HSD17B2 transcriptional levels in early stages, including polyps, underscores its interest as a candidate gene for further study in early CRC detection. Further research on the role of these enzymes, particularly in the context of the interconversion of estrone and estradiol, could provide valuable insights into the progression of CRC and potential therapeutic targets. In this regard, AKR1C3 is also of interest as it intriguingly performs the opposite function of HSD17B2. Although its transcriptional patterns are not as robust in metastasis, it still shows one of the most significant expression changes that remain consistent across all studies.

**Figure 3:**
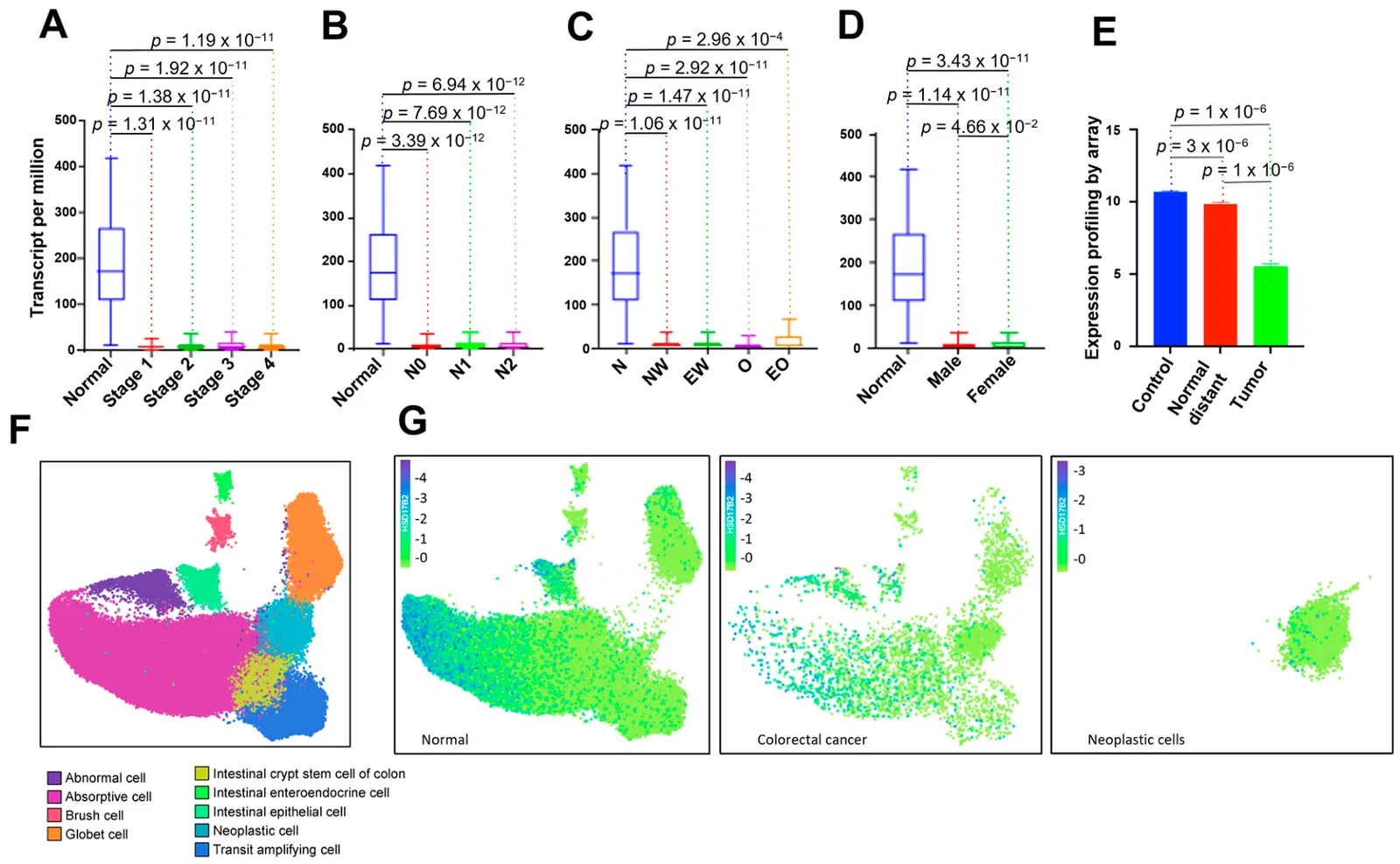
Expression profile and cellular localization of HSD17B2 in colon adenocarcinoma through different comparisons. (**A**–**E**) Transcriptional expression levels (TPM) of *HSD17B2* stratified by (**A**) individual cancer stage, (**B**) nodal metastasis status, (**C**) patient weight/obesity status, (**D**) sex and (**E**) distance from the tumor. (**F**) Single-cell expression pattern of *HSD17B2* and (**G**) its relative expression profile across identified cell lineages. Statistical significance was calculated using the default parameters implemented in UALCAN or Student’s *t*-test for GEO-derived data with exact *p*-values provided for each comparison.

Given its robust and reproducible expression changes across all analyses, we decided to focus on the HSD17B2 enzyme for a more in-depth investigation (Figure 3). Data from 114 controls and 1097 tumor samples from the UALCAN database were used to perform this subsequent analysis.

The expression pattern shown in Figure 3A is consistent with the results independently observed in both GEO cohorts (GSE40967 and GSE41258), supporting the reproducibility of the *HSD17B2* expression pattern across different transcriptomic datasets. They showed a clear decline in *HSD17B2* expression as cancer advanced from normal tissue to stage IV. Similarly, the nodal metastasis status (Figure 3B) indicated lower expression of *HSD17B2* in samples with positive nodal involvement (*p* = 6.94 × 10^−12^).

Furthermore, patient weight (Figure 3C) also appeared to correlate with gene expression levels, with higher body weight showing reduced *HSD17B2* levels (*p* = 2.96 × 10^−4^). Moreover, sex differences (Figure 3D) were apparent, as women had significantly higher expression compared to men (*p* = 3.43 × 10^−11^), which again points to potential underlying sex-related factors in CRC.

When analyzing expression levels in primary tumors and adjacent normal tissues (Figure 3E), controls showed the highest levels of *HSD17B2*, which decreased in tumor samples (*p* = 1 × 10^−6^). Further analysis of tumor position within the colon relative to normal tissue confirmed significant differences (*p* < 1 × 10^−6^), with tumor tissues showing reduced expression, in addition to lower expression of *HSD17B2* in left-sided tumors, a location often associated with worse prognosis.

Importantly, we evaluated *HSD17B2* expression at the single-cell level (Figure 3F), identifying distinct clusters. The analysis of expression patterns by cell type (Figure 3G) demonstrated that neoplastic cells had markedly lower expression of *HSD17B2* compared to normal cells, further supporting its potential involvement in colon tumorigenesis.

These results suggest that *HSD17B2* downregulation is associated with advanced disease stages, metastatic potential, and poorer outcomes, underscoring its potential as a candidate biomarker for early detection, prognosis and disease progression in CRC.

### 2.4. Receiver Operating Characteristic (ROC) Curve Analysis of HSD17B2 and Family Enzyme Combinations as Potential CRC Biomarkers

**Figure 4:**
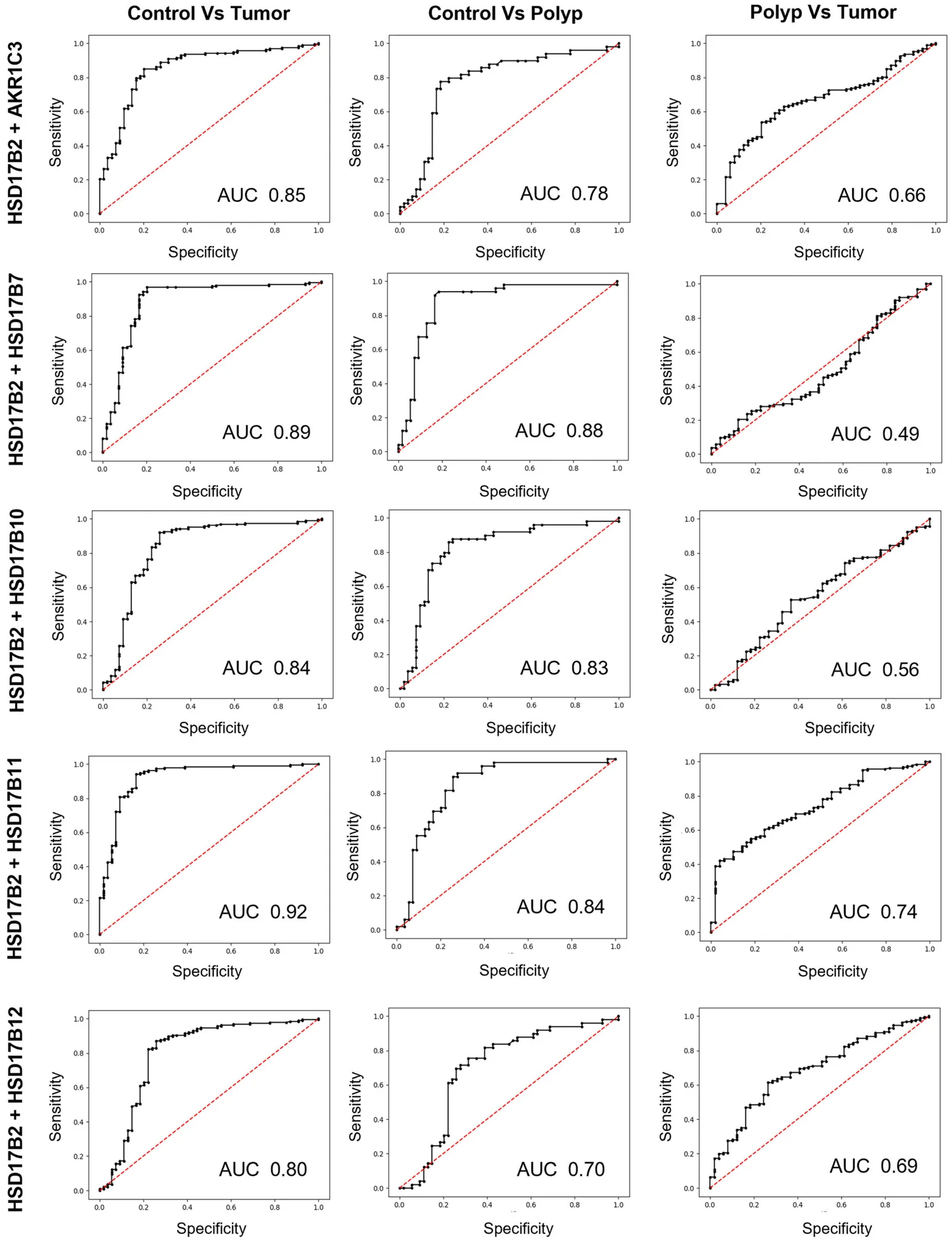
Diagnostic performance of HSD17B2 and selected HSD17B enzymes across CRC progression stages. Receiver Operating Characteristic (ROC) curves illustrating the discriminatory capacity of HSD17B enzymes across three clinical comparisons (organized in columns): control vs. tumor (**left**), control vs. polyp (**middle**), and polyp vs. tumor (**right**). Black line in each panel represents the ROC curve for the combined model of the two evaluated enzymes, while the red dashed line indicates the non-discrimination reference line. Sensitivity and specificity values were derived from logistic regression analyses, and the Area Under the Curve (AUC) is provided as a metric to assess the classification performance in each case. ROC analyses were performed using logistic regression.

To further evaluate this potential role of HSD17B2 and other enzymes in the HSD17B family as candidate biomarkers for distinguishing between control, polyp, and tumor samples, ROC curve analyses were conducted. The comparisons included Control vs. Tumor, Control vs. Polyp, and Polyp vs. Tumor (Figure 4).

The combination of HSD17B2 with AKR1C3 yielded an Area Under the Curve (AUC) of 0.85, indicating strong discriminatory power in the first two scenarios. This pattern suggests that the HSD17B2 + AKR1C3 combination warrants further study as a potential panel to assist in differentiating between control and tumor samples, as well as control and polyp, despite showing lower discriminatory ability when distinguishing between polyps and tumors.

Similarly, the combination of HSD17B2 with HSD17B7 demonstrated excellent performance in the first two comparisons, with AUC values around 0.88, indicating a high capacity to differentiate between control and tumor, as well as control and polyp. However, the performance was notably weaker for the polyp vs. tumor comparison, suggesting that HSD17B2 + HSD17B7 may be less informative for distinguishing between polyps and tumors.

The HSD17B2 + HSD17B10 combination showed solid performance in distinguishing control samples from both tumors and polyps, but its ability to differentiate between polyps and tumors was relatively modest.

Meanwhile, HSD17B2 + HSD17B11 exhibited the best performance among the analyzed combinations, with AUC values of 0.92, 0.84, and 0.74 for control vs. tumor, control vs. polyp, and polyp vs. tumor, respectively. This combination demonstrated the most effective discriminatory power in this dataset, particularly in identifying tumor samples, and stands out as a promising candidate panel for differentiating between the three groups.

Lastly, we tested the combination of HSD17B2 with HSD17B12. While the discriminatory capacity was moderate for the first two comparisons, it was slightly lower for the polyp vs. tumor comparison, suggesting a more limited ability to distinguish between polyps and tumors.

These results indicate that HSD17B2, when combined with specific members of the HSD17B family, holds promise as a candidate biomarker panel, particularly for distinguishing tumor and control tissue. The combination of HSD17B2 + HSD17B11 showed the strongest discriminatory power across all comparisons, especially in identifying adenocarcinomas.

This ability to distinguish between stages could support further exploratory work towards identifying malignant lesions, potentially contributing to future strategies for CRC risk stratification.

### 2.5. Prognostic Significance of HSD17B Family Expression in Colorectal Cancer: Overall (OS) and Relapse-Free Survival (RFS)

Besides this potential application, we have also investigated the impact of the HSD17B family members on CRC progression. The Kaplan–Meier survival analysis demonstrates that the expression levels of several HSD17B family genes are significantly associated with both OS and RFS in patients.

**Figure 5:**
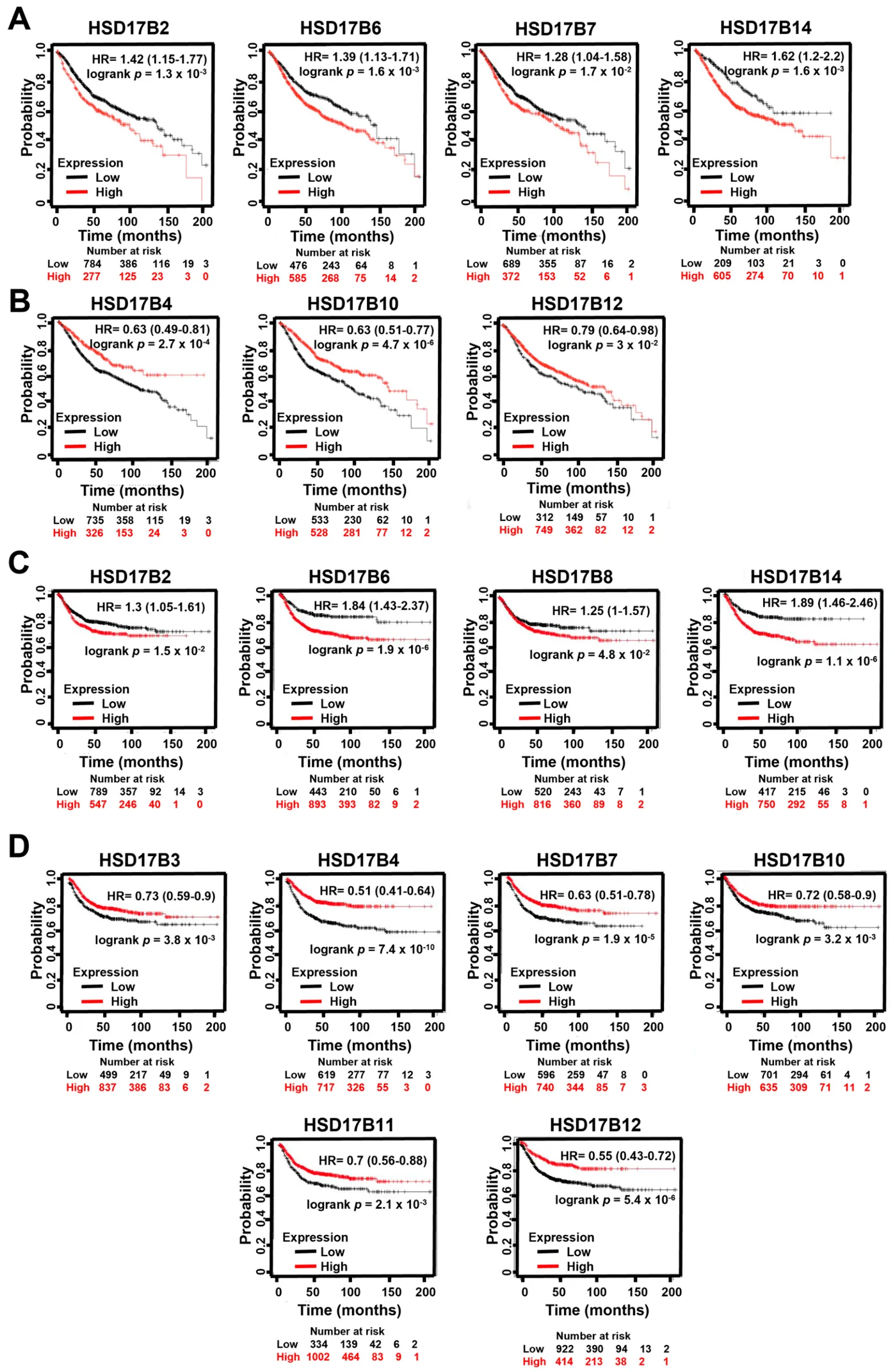
Prognostic value of *HSD17B* family gene expression in colon adenocarcinoma (Kaplan–Meier survival analyses). (**A**,**B**) OS curves stratified by gene expression levels, highlighting (**A**) genes where high expression is associated with poor prognosis and (**B**) genes where low expression correlates with poor outcomes. (**C**,**D**) + RFS curves categorized by (**C**) high expression levels predicting reduced RFS and (**D**) low expression levels linked to higher recurrence risk. Survival analyses were generated using the Kaplan–Meier method with log-rank test.

In the OS analysis (Figure 5A,B), higher expression of certain genes such as *HSD17B2*, *HSD17B6*, *HSD17B7*, and *HSD17B14* was associated with a worse prognosis, as indicated by reduced survival probabilities over time (Figure 5A). Conversely, lower expression of other genes such as *HSD17B4*, *HSD17B10*, and *HSD17B12* correlated with poorer outcomes, indicating that higher expression of these genes may be associated with a favorable prognostic profile (Figure 5B).

Similarly, the RFS analysis (Figure 5C,D) reveals that a high expression of genes such as *HSD17B2*, *HSD17B6*, *HSD17B8*, and *HSD17B14* is associated with reduced relapse-free survival (Figure 5C). On the other hand, lower expression of genes like *HSD17B3*, *HSD17B4*, *HSD17B7*, *HSD17B10*, *HSD17B11*, and *HSD17B12* significantly correlates with worse RFS, suggesting that higher transcript levels of these genes may align with a lower likelihood of recurrence (Figure 5D).

In comparing the results for OS and RFS, a clear pattern emerges regarding the prognostic impact of *HSD17B* gene expression. For both OS and RFS, several genes, such as *HSD17B2*, *HSD17B6*, and *HSD17B14*, are consistently associated with worse outcomes when expressed at higher levels, suggesting that their higher expression levels are linked to both a decrease in overall patient survival and an increased likelihood of relapse.

On the other hand, genes such as *HSD17B3*, *HSD17B4*, *HSD17B7*, *HSD17B10*, and *HSD17B12* are consistently associated with favorable outcomes in both OS and RFS when expressed at higher levels, as their lower expression is linked to a poorer prognosis in both survival metrics. This consistency across OS and RFS highlights the dual importance of certain HSD17B family members not only in evaluating overall mortality risk but also in influencing the risk of disease recurrence, underscoring their potential as candidate prognostic biomarkers and subjects for future research in CRC management.

## 3. Discussion

The involvement of estrogen metabolism in CRC has gained significant attention due to its implications in tumorigenesis [11,13,25]. Among the enzymes regulating the balance between estradiol and estrone—two key estrogens with distinct biological effects [9,16]—HSD17B2 emerges as a critical player. In our study, we identified consistent alterations in the expression of several enzymes, including HSD17B2, AKR1C3, HSD17B7, HSD17B10, HSD17B11, and HSD17B12, across various stages of CRC. Notably, HSD17B2 displayed the most pronounced changes even in precancerous lesions such as polyps, pointing to its potential role in early tumorigenesis. Importantly, the differential expression pattern of *HSD17B2* was consistently observed across the TCGA cohort and two independent GEO datasets, supporting the robustness and reproducibility of these findings in different patient cohorts and transcriptomic platforms.

HSD17B2 is responsible for converting estradiol into estrone [26,27]. This enzymatic activity is crucial in tissues like the colon, where the estrogen balance influences cell proliferation and cancer progression [28,29]. Our findings, along with prior studies, indicate that *HSD17B2* is often downregulated in CRC, which could theoretically lead to increased local estradiol levels [30]. Elevated estradiol, known for its proliferative effects, may foster a tumor-promoting microenvironment, particularly in estrogen-sensitive contexts [31,32]. These observations are consistent with previous studies, suggesting that reduced *HSD17B2* expression could contribute to CRC progression through alterations in estrogen metabolism [33]. This aligns with evidence linking estrogenic imbalances to CRC progression, especially in postmenopausal women, who generally exhibit higher levels of estrone than estradiol [34].

Interestingly, while *HSD17B2* is commonly downregulated in CRC [35,36], our KM Plotter data show that its higher expression is paradoxically associated with poorer prognoses. This suggests that estrone, traditionally considered a weaker estrogen [37], might play a more significant role in CRC progression than previously thought. Elevated *HSD17B2* levels—and, by extension, potentially higher local estrone levels—have been linked to more aggressive disease stages and worse survival outcomes in our KM-Plotter analysis. The higher incidence of CRC in postmenopausal women—characterized by elevated estrone relative to estradiol—further supports this hypothesis [38]. Moreover, studies in other cancers suggest that estrone could promote tumor growth even in cancers not primarily driven by estrogen receptors [39]. These findings challenge the assumption that estrone is biologically inert in CRC, underscoring its potential relevance in tumor progression. In line with this, recent studies have emphasized the complex and context-dependent role of estrogens in CRC, where estradiol may exert both protective and tumor-promoting effects depending on the molecular and cellular environment [40].

In addition to HSD17B2, we observed significant expression changes in other estrogen-metabolizing enzymes. For instance, *AKR1C3*, which contributes to estradiol production, is frequently downregulated in CRC, potentially reducing local estradiol levels. Conversely, the higher expression of *HSD17B7* and moderate upregulation of *HSD17B12* might increase estradiol availability, complicating predictions about the estrogenic status in the tumor microenvironment. The upregulation of *HSD17B10* and the overexpression of *HSD17B7* further contribute to the complexity of the hormonal landscape, underscoring the intricate regulation of estrogen metabolism in CRC.

Serum hormone analyses from previous studies provide additional insights. Although no significant differences in estradiol levels were observed between CRC patients and healthy controls, a slight increase in estrone levels was noted in CRC patients, although without statistical significance [41]. Additionally, higher total testosterone levels were consistently reported in female CRC patients [6], suggesting broader hormonal disruptions. These observations further support the hypothesis that estrone, rather than estradiol, could play a prominent role in CRC progression.

The dual role of HSD17B2 in CRC, characterized by early downregulation in precancerous states and higher expression in advanced disease, suggests its potential relevance as a biomarker for early stratification and prognosis.

The early downregulation of *HSD17B2*, particularly in polyps, supports its potential utility in identifying precancerous lesions. This early decrease in expression suggests that alterations in HSD17B2 may occur at initial stages of tumorigenesis, potentially reflecting early disruptions in estrogen metabolism within the colonic epithelium. Given the role of HSD17B2 in regulating the balance between estradiol and estrone, its downregulation could contribute to a microenvironment that favors tumor initiation and progression. From a clinical perspective, these observations reinforce its interest as a candidate marker, as identifying such molecular alterations at the polyp stage could help inform strategies to monitor early neoplastic transformations.

Beyond its role in early stages, the association of HSD17B2 expression with clinical outcomes suggests its potential prognostic value. In this context, numerous studies have demonstrated that molecular genetic parameters, including gene expression profiles, mutational status, and transcriptomic signatures, can serve as prognostic biomarkers in cCRC, providing valuable information on patient survival and disease progression [42]. Established examples include mutations in key oncogenes and tumor suppressors, as well as gene expression-based classifiers that stratify patients into distinct prognostic subgroups [43,44,45]. In line with these findings, the expression pattern of HSD17B2 observed in our study shares key features with these molecular biomarkers, particularly those based on gene expression dynamics. However, unlike many established markers primarily linked to tumor-intrinsic pathways such as proliferation or genomic instability, HSD17B2 may reflect alterations in local hormone metabolism, highlighting a complementary and less explored biological axis in CRC progression.

Interestingly, as shown in the ROC analysis, the combination of HSD17B2 and HSD17B11 showed the highest discriminatory power in distinguishing CRC from benign conditions and precancerous lesions in our dataset. This finding suggests that integrating multiple biomarkers from the HSD17B family could enhance diagnostic precision, particularly for detecting CRC at its earliest stages. In line with this, recent studies have highlighted both the tumor-suppressive role of HSD17B2 and the complex, context-dependent effects of estrogen signaling in colorectal tumorigenesis, reinforcing the biological plausibility of these findings [33,40]. Altogether, these results support the relevance of estrogen metabolism-related pathways in CRC and point toward the potential clinical utility of HSD17B2 not only as an individual biomarker, but also as part of multi-marker strategies for early risk stratification and prognostic evaluation.

Nevertheless, several limitations of this study should be acknowledged. First, our findings are based on retrospective bioinformatic analyses of publicly available datasets, which carry inherent limitations regarding clinical annotation and cohort heterogeneity. Second, while gene expression levels provided robust prognostic and diagnostic insights, these findings rely on transcriptomic data; direct quantification of enzyme protein levels and local tissue concentrations of estrone and estradiol was not performed. Finally, the biological mechanisms proposed herein warrant further experimental and functional validation in vitro and in vivo to definitively confirm the causal roles of these enzymes in CRC progression and validate their utility in larger, well-characterized prospective cohorts.

## 4. Materials and Methods

### 4.1. Expression and Methylation Levels

In this study, we performed a comprehensive evaluation of the expression and methylation levels of the genes encoding our study enzymes and the total protein expression; a search was performed in 2024 using The University of Alabama at Birmingham Cancer data analysis Portal (UALCAN) (URL: https://ualcan.path.uab.edu/index.html. Accessed on 10 October 2025).

Expression and methylation levels of the genes and their statistical significance were obtained from The Cancer Genome Atlas (TCGA). For this search, each gene of interest was queried in the colon adenocarcinoma (COAD) TCGA dataset. Expression levels were analysed based on sample types, individual cancer stages, patients’ gender, weight, nodal metastasis status and TP53 mutation status, while methylation levels were only evaluated based on sample types.

For survival analyses, patients were stratified into expression groups according to the default settings of the analysis platform, in which samples are categorized based on the distribution of gene expression values within the cohort rather than using a predefined absolute cutoff value.

Total protein expression data and their statistical significance were obtained from the Clinical Proteomic Tumor Analysis Consortium (CPTAC) and the International Cancer Proteogenome Consortium (ICPC) datasets. Total protein expression levels were analysed based on sample types for each enzyme.

### 4.2. Survival Analysis

The objective of this study was to analyse the prognosis effects of mRNA expression of the 14 enzymes in colon adenocarcinoma patients. The Kaplan–Meier Plotter (KM Plotter) (URL: https://kmplot.com/analysis/. Accessed on 12 November 2025) was used to perform prognosis analysis between the mRNA expression level of the selected genes analysed by gene chip. In this search, patients were split by “Auto select best cutoff” and no restrictions were added. The analysis was performed for OS, with *n* = 1061; RFS with *n* = 1336; and PPS (Post-progression survival), with *n* = 311. Obtained graphs show patients’ survival probability in months based on the high or low expression of each enzyme.

### 4.3. GEO Datasets Search

To evaluate the reproducibility of the findings obtained from the TCGA dataset, two independent Gene Expression Omnibus (GEO) cohorts were selected. A search of terms related with colorectal cancer and metastases was performed in the Gene Expression Omnibus (GEO) Datasets (URL: https://www.ncbi.nlm.nih.gov/gds. Accessed on 25 October 2025) with the study subtype Expression profiling by array. Datasets with the highest of patients including the 14 enzymes in the study were chosen. The datasets GSE40967, GSE41258 and GSE44076 were evaluated with GEO2R and downloaded. GraphPad Prism Software version 8.0.0 (GraphPad Software, San Diego, CA, USA) was used to process data. Data obtained show enzyme expressions in each patient or healthy donor.

All data included in this study were retrieved from databases; therefore, informed consent and ethics approval were not needed.

### 4.4. Single-Cell Transcriptomic Analysis

scRNA-seq data were sourced from a publicly available dataset comprising 63 specimens, including 32 tumor samples (PMID: 34910928). The dataset is part of the Human Tumor Atlas Network (HTAN) Consortium and is accessible via the HTAN Data Portal (https://data.humantumoratlas.org/. Accessed on 3 December 2025). Data is available at the Human Cell Atlas Project (https://explore.data.humancellatlas.org/projects/50154d1e-2308-44bf-9608-10c7afaa560b. Accessed on 14 December 2025) and the dbGaP Study Accession (phs002371.v6.p1). The cells were encapsulated using a modified inDrop platform, and sequencing libraries were prepared following the TruDrop protocol. The annotated scRNA-seq data were analyzed using Cellxgene (v1.1.1), a tool designed for interactive exploration of single-cell data. Uniform Manifold Approximation and Projection (UMAP) embeddings were generated within Cellxgene to visualize the cellular landscape (https://doi.org/10.1101/2020.08.28.270652).

### 4.5. ROC Analysis

A logistic regression model was employed to determine the relationship between the sum of enzyme expression levels (independent variable) and the presence or absence of disease (dependent variable) from the dataset GSE41258 obtained from GEO2R database. The model was fitted using the LogisticRegression module from the sklearn package in Python version 3.10.0 (Python Software Foundation, Wilmington, DE, USA), with the default optimization algorithm lbfgs.

After fitting the logistic regression model, probabilities for each sample belonging to the diseased (positive) or non-diseased (negative) class were calculated. The probability of belonging to the positive class was extracted and used as input for subsequent analysis.

The roc_curve function from the sklearn package was employed to calculate the false positive rate (1−Specificity) and true positive rate (Sensitivity) for each threshold. These metrics are fundamental for constructing the Receiver Operating Characteristic (ROC) curve.

The ROC curve was plotted using the pyplot module from the matplotlib library to provide a visual representation of the model’s performance.

### 4.6. Statistics

Data are expressed as mean ± SEM. Differences between groups of data obtained from GEO datasets were analysed using multiple Student’s *t*-tests for pairwise comparisons.

For analyses derived from publicly available databases, statistical significance and *p*-values were calculated using the methods implemented within each platform. Specifically, for gene expression and methylation analyses performed using UALCAN, comparisons between two groups were conducted using Student’s *t*-test, while comparisons involving multiple groups were performed using one-way ANOVA, as provided by the platform.

Survival analyses performed using Kaplan–Meier Plotter were based on Kaplan–Meier curves, and statistical significance was evaluated using the log-rank test.

A *p*-value < 0.05 was considered statistically significant. Statistical analysis of GEO-derived data was performed using GraphPad Prism software.

## 5. Conclusions

Our findings, consistently observed across TCGA and two independent GEO cohorts, underscore the complexity of estrogen metabolism in CRC and the central role of HSD17B2 in this process. Its dual behavior—as an enzyme downregulated in early stages but elevated in advanced disease—positions it as a dynamic candidate biomarker and potential target for therapeutic investigation. Furthermore, the emerging role of estrone in CRC progression challenges traditional perspectives on estrogen biology, suggesting that pathways involving this hormone warrant further investigation.

Future studies should aim to elucidate the precise mechanisms through which HSD17B2 and other HSD17B enzymes influence CRC progression. This could involve exploring their interactions with the tumor microenvironment, analyzing estrone’s specific effects on colorectal tissues, and evaluating therapeutic approaches to modulate these pathways. By advancing our understanding of estrogen metabolism in CRC, we can pave the way for innovative diagnostic, prognostic, and treatment strategies that could ultimately contribute to improving patient care.

## Data Availability

The original contributions presented in this study are included in the article. Further inquiries can be directed to the corresponding authors.

## Supplementary Materials

The following supporting information can be downloaded at: https://www.mdpi.com/article/10.3390/ijms27177614/s1.

## Abbreviations

CRC	Colorectal Cancer
HSD17B	Hydroxysteroid (17-beta) dehydrogenase
TCGA	The Cancer Genome Atlas
COAD	Colon Adenocarcinoma
UALCAN CPTAC	The University of Alabama at Birmingham Cancer data analysis Portal Clinical Clinical Proteomic Tumor Analysis Consortium
ICPC	International Cancer Proteogenome Consortium
KM Plotter	Kaplan–Meier Plotter
OS	Overall Survival
RFS	Relapse-Free Survival
GEO	Gene Expression Omnibus
UMAP	Uniform Manifold Approximation and Projection
ROC	Receiver Operating Characteristic
TPM	Transcript per million
PT	Primary tumor
C	Control
NW	Normal Weight
OW	Overweight
O	Obese
EO	Extreme Obese
HTAN	Human Tumor Atlas Network
