# Supplementary material for: Exploring the Role of HSD17B2 in Colorectal Cancer Through Bioinformatic Analysis: Preliminary Insights for Prognostic Evaluation
Source: Int J Mol Sci. 2026 Aug 25;27(17):7614. doi: 10.3390/ijms27177614 (PMC13566651; doi:10.3390/ijms27177614)
Supplement: Supplementary file 1 [file ijms-27-07614-s001.zip › ijms-4446924-supplementary.pdf]

Enzyme	Catalysed reaction	Reference
HSD17B1	Estrone + NAD(P)H + H+ → 17β-estradiol + NAD(P)+	[25,26]
	Androstenedione + NAD(P)H + H^+ → Testosterone + NAD(P)$^+$	
	Androstenediol + NADH + H^+ → Dehydroepiandrosterone + NAD$^+$	
	Dihydrotestosterone + NADH + H+ → 3β-diol / 3α-diol + NAD+	
HSD17B2	17β-estradiol + NAD(P)+ → Estrone + NAD(P)H + H+	[27–29]
	Testosterone + NAD$^+$ → Androstenedione + NADH + H^+	
	Dihydrotestosterone + NAD(P)$^+$ → Androstanedione + NAD(P)H + H^+	
	Androstenediol + NADH + H^+ → Dehydroepiandrosterone + NAD$^+$	
	20 α-dihydroprogesteron + NAD+ → Progesterone + NADH + H+	
HSD17B3	Androstenedione + NADPH + H^+ → Testosterone + NADP$^+$	[30]
HSD17B4	17β-estradiol + NAD(P)$^+$ → Estrone + NAD(P)H + H^+	[31]
	Testosterone + NAD$^+$ → Androstenedione + NADH + H^+	
	Androstenediol + NADH + H^+ → Dehydroepiandrosterone + NAD$^+$	
	Dihydrotestosterone + NAD(P)$^+$ → Androstanedione + NAD(P)H + H^+	
AKR1C3 /HSD17B5	Dehydroepiandrosterone + NAD$^+$ → Androstenediol + NADH + H^+	[32,33]
	Androstenedione + NADPH + H^+ → Testosterone + NADP$^+$	
	Androstanedione + NAD(P)H + H^+ → Dihydrotestosterone + NAD(P)$^+$	
	Androsterone + NAD+ → Androstanediol + NADH + H^+	
	Estrone + NAD(P)H + H^+ → 17β-estradiol + NAD(P)$^+$	
HSD17B6	Dihydrotestosterone + NADH + H^+ → 3β-diol / 3α-diol + NAD$^+$	[34,35]
	17β-estradiol + NAD(P)$^+$ → Estrone + NAD(P)H + H^+	
	Androsterone + NAD+ → Epi-androsterone + NADH + H^+	
	All-trans-retinol + NAD+ → All-trans-retinal + H^+ + NADH	
	Testosterone + NAD+ → Androstenedione + NADH + H+	
	3α/β-hydroxy-5α-pregnan-20-one + NAD+ → 5α-pregnane-3,20-dione + H^+ + NADH	
HSD17B7	Estrone + NAD(P)H + H^+ → 17β-estradiol + NAD(P)$^+$	[36]
	Dihydrotestosterone + NADH + H^+ → 3β-diol / 3α-diol + NAD$^+$	
HSD17B8	17β-estradiol + NAD$^+$ → Estrone + NADH + H^+	[37]
HSD17B9	-	
HSD17B10	17β-estradiol + NAD$^+$ → Estrone + NADH + H^+	[38]
	Allopregnanolone + NAD$^+$ → 5α-dihydroprogesterone + NADH + H^+	
	2-methyl-3-hydroxybutyryl-CoA + NAD+ ↔ 2-methyl-acetoacetyl-CoA + NADH + H+	
HSD17B11	17β-estradiol + NAD(P)$^+$ → Estrone + NAD(P)H + H^+	[39]
HSD17B12	Estrone + NAD(P)H + H^+ → 17β-estradiol + NAD(P)$^+$	[40]
HSD17B13	-	
HSD17B14	17β-estradiol + NAD(P)$^+$ → Estrone + NAD(P)H + H^+	[41]
	Androstenediol + NADH + H^+ → Dehydroepiandrosterone + NAD$^+$	
Aromatase (CYP19A1)	Testosterone + NADPH + H+ ↔ 17β-estradiol + NADP	[42,43]
	Androstenedione + NADPH + H+ ↔ Estrone + NADP+	
